# Big data, small explanatory and predictive power: Lessons from random forest modeling of on-farm yield variability and implications for data-driven agronomy

**DOI:** 10.1016/j.fcr.2023.109063

**Published:** 2023-10-15

**Authors:** João Vasco Silva, Joost van Heerwaarden, Pytrik Reidsma, Alice G. Laborte, Kindie Tesfaye, Martin K. van Ittersum

**Affiliations:** a Sustainable Agrifood Systems, CIMMYT, Harare, Zimbabwe; b Plant Production Systems, Wageningen University, Wageningen, the Netherlands; c Sustainable Impact Department, IRRI, Los Baños, the Philippines; d Sustainable Agrifood Systems, CIMMYT, Addis Ababa, Ethiopia

**Keywords:** Sustainable intensification, Model accuracy, Model precision, Linear mixed models, Machine learning

## Abstract

**Context:**

Collection and analysis of large volumes of on-farm production data are widely seen as key to understanding yield variability among farmers and improving resource-use efficiency.

**Objective:**

The aim of this study was to assess the performance of statistical and machine learning methods to explain and predict crop yield across thousands of farmers’ fields in contrasting farming systems worldwide.

**Methods:**

A large database of 10,940 field-year combinations from three countries in different stages of agricultural intensification was analyzed. Random effects models were used to partition crop yield variability and random forest models were used to explain and predict crop yield within a cross-validation scheme with data re-sampling over space and time.

**Results:**

Yield variability in relative terms was smallest for wheat and barley in the Netherlands and for wheat in Ethiopia, intermediate for rice in the Philippines, and greatest for maize in Ethiopia. Random forest models comprising a total of 87 variables explained a maximum of 65 % of cereal yield variability in the Netherlands and less than 45 % of cereal yield variability in Ethiopia and in the Philippines. Crop management related variables were important to explain and predict cereal yields in Ethiopia, while predictive (i.e., known before the growing season) climatic variables and explanatory (i.e., known during or after the growing season) climatic variables were most important to explain and predict cereal yield variability in the Philippines and in the Netherlands, respectively. Finally, model cross-validation for regions or years not seen during model training reduced the R^2^ considerably for most crop x country combinations, while for wheat in the Netherlands this was model dependent.

**Conclusion:**

Big data from farmers’ fields is useful to explain on-farm yield variability to some extent, but not to predict it across time and space.

**Significance:**

The results call for moderate expectations towards big data and machine learning in agronomic studies, particularly for smallholder farms in the tropics where model performance was poorest independently of the variables considered and the cross-validation scheme used.

## Highlights

• Big data is an important asset for agronomic research and decisionmaking.
• Random forest was used to explain and predict yield variability across farmers’ fields.
• 87 variables explained a maximum of 65 % of yield variability.
• Model cross-validation over space and time reduced model performance considerably.
• Big data is useful to explain yield variability to some extent, but less so to predict it.

## 1 Introduction

Since the advent of precision farming, it has become clear that data are an important asset for agronomic research and decision making (Wolfert et al., 2017). The increasing availability of large volumes of high-resolution biophysical data (Hengl et al., 2017, Funk et al., 2015), combined with geo-referenced farmer’s field data, has created opportunities for a data-driven agronomy across wide geographic scales and at relatively little cost (Nayak et al., 2022a, Silva et al., 2020b, Cui et al., 2018, Rattalino Edreira et al., 2017, Frelat et al., 2016). Such wealth of information is expected to foster an agronomic revolution (Vanlauwe & Dobermann, 2020) and to accelerate the sustainable intensification of crop production (Cassman and Grassini, 2020). This could not be more timely given the grand challenges crop production will be facing in the coming decades: ensuring food and nutrition security in light of climate change while avoiding conversion of natural habitats and biodiversity loss (Silva and Giller, 2020).

’Big data’ in the context of this paper refers to observational datasets typically considered for data-driven approaches in agricultural research, regardless of the actual volumes of data involved (see de Mauro et al., 2016 for a formal definition). The most direct application of big data in agriculture is in explaining and/or predicting crop yield variability in farmers’ fields across time and space. This is a daunting challenge given the large number of interacting factors contributing to crop yield variability (van Klompenburg et al., 2020, Beza et al., 2017, Ronner et al., 2016). Successful prediction of yield variability may help agronomists’ and farmers’ understanding and decision making. Moreover, systematic patterns in yield variability can be further translated into decision-support tools for different stakeholders, thus contributing to evidence-based investments in research and development programs. Such applications require quantitative approaches capable of dealing with a large number of interacting variables. Machine learning methods operate at the intersection between computer science and statistics (Hey et al., 2009) and have been shown successful in finding predictive relationships in complex data sets over a wide range of applications, also in the agricultural sector (e.g., Paudel et al., 2021, Tseng et al., 2021, van Klompenburg et al., 2020).

The usefulness of big data analytics may differ for different farming systems worldwide, depending on their level of intensification and on the biophysical and socio-economic context in which they operate (Silva et al., 2021b). Different farming systems most likely also differ in environmental conditions and yield variability as well as in the availability of biophysical and agronomic data. Poor data quality and availability, for instance, is a recurrent issue for smallholder farming systems in sub-Saharan Africa (e.g., Carletto et al., 2013) and leads to unsatisfactory predictions of crop yield and response to nutrients (van Heerwaarden, 2022, Ronner et al., 2016). Conversely, data availability is generally better in high-yielding farming systems, but even there yield prediction is far from perfect (Mulders et al., 2021, Silva et al., 2020b). However, there has not been to date any systematic comparison of the ability to explain and predict crop yield variability on-farm data from farming systems covering different biophysical conditions and stages of intensification.

The objective of this study was to assess the potential for typical on-farm production data from cereal crops in different geographic regions to uncover systematic and predictable patterns in yield variation. We evaluated the partitioning of yield variation in space and time and quantified the amount of farm-level variability that could be accounted for by external agronomic and biophysical variables. An explicit distinction was made between predictive variables, which are known prior (a priori) to a given growing season, and explanatory variables which are only known during or after (ex-post) the growing season (van Heerwaarden et al., 2023). We hypothesize that explanatory variables account for more variation in crop yield than predictive variables and that model explanatory and predictive power decrease when extrapolating in space and time. A large database of farmer field data was compiled for maize and wheat in Ethiopia, rice in the Philippines, and winter wheat and spring barley in the Netherlands, comprising primary, farmer reported, crop management and production data and secondary spatially explicit weather, climate and soil data. The analysis contributes to a growing body of literature on machine learning applications in agronomy and to the analysis of prospects offered by big data to achieve sustainable intensification of crop production in the future.

## 2 Analytical framework

**Fig. 1:**
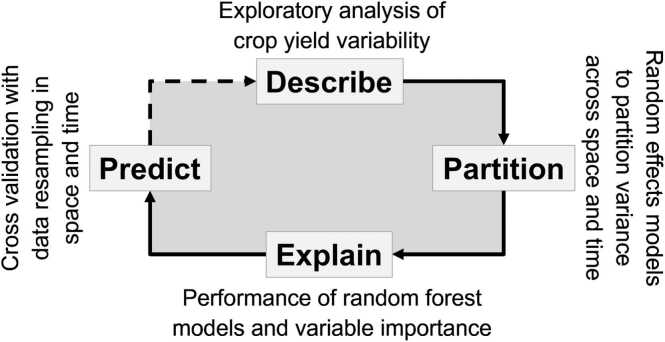
Analytical framework adopted to explain and predict crop yield variability over space and time. Data analyses build upon linear mixed models with random effects to partition residual variance in crop yield and upon random forest to explain and predict crop yield based on a large set of covariates.

Our framework for explaining and predicting yield variability in space and time comprised four steps (Fig. 1). First, variability of farmer reported yields was described through an exploratory analysis using boxplots and scatterplots of mean crop yield and the respective standard deviation across unique year × district combinations. Second, a random effects model was used to partition yield variability among different sources of spatial and temporal variation, separating within-farm residual variation from systematic sources of variation as represented by different temporal (year) and spatial scales (province, district, farm). Third, random forest models, incorporating a large set of covariates, obtained through household surveys and high-resolution spatial databases were fitted to the data to account for as much yield variability as possible. Variable importance was computed to identify the key biophysical and crop management drivers of yield variability and statistical metrics were used to evaluate the accuracy and precision of the fitted models. A distinction was made between predictive and explanatory variables, noting the difference in ability to explain yield variability after the growing season as compared to predicting yield at the start or during the growing season. All time-invariant variables were identified as *predictive variables*, as they can be known ahead of any growing season. Conversely, *explanatory variables* were identified as those which are specific to a given growing season, which may explain yield variability in that specific season but do not contribute to predicting future outcomes. Finally, a cross-validation scheme with data re-sampling over space and time was employed to evaluate the goodness-of fit of random forest models when extrapolated to newly sampled locations or seasons.

## 3 Materials and methods

### 3.1 Database of farm field data

#### 3.1.1 Description of data sets

The database analysed here comprised a total of 10,940 geo-referenced field × year observations: 7220 observations from Ethiopia, 1960 observations from the Philippines, and 1760 observations from the Netherlands (Table 1). These data were obtained through household surveys in Ethiopia and the Philippines and through commercial software systems in the Netherlands and were previously used for yield gap decomposition (Silva et al., 2021a, Assefa et al., 2020) or resource-use efficiency assessments (Silva et al., 2020, 2018). Historical weather data for different sites in each country are provided in Supplementary Figure 1.

**Fig. 2:**
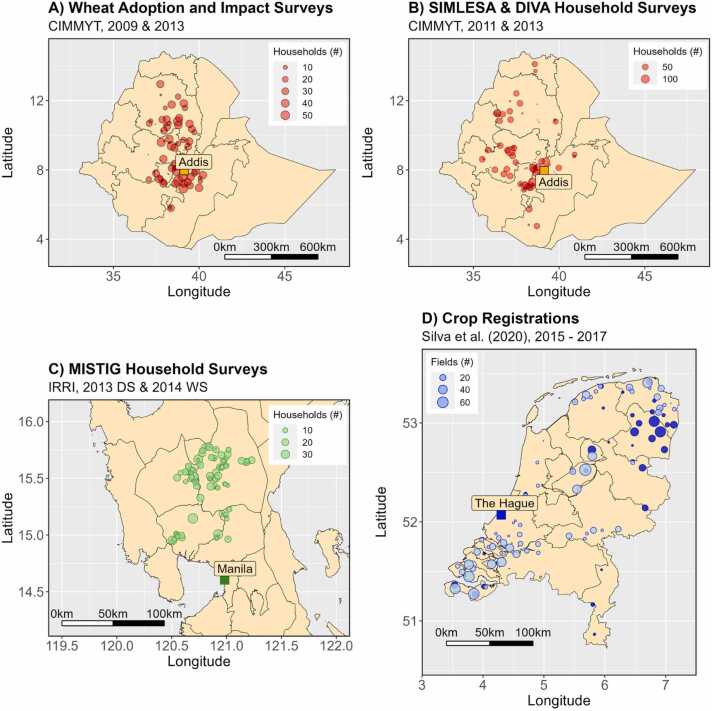
Location of the farms and fields surveyed and analysed in this study: (A) wheat in Ethiopia during the Meher seasons of 2009 and 2013, (B) maize in Ethiopia during the Meher seasons of 2010 and 2013, (C) lowland irrigated rice during the 2013 dry season (DS) and 2014 wet season (WS) in Central Luzon, Philippines, and (D) winter wheat (light blue) and spring barley (dark blue) in the Netherlands during the period 2015–2017. See text for further information about the SIMLESA, DIVA, and MISTIG projects, which the data were collected.

**Table 1:** Descriptive statistics of selected variables for wheat and maize crops in Ethiopia (ETH), wet season (WS) and dry season (DS) rice crops in the Philippines (PHL), and wheat and barley crops in the Netherlands (NLD). Aridity index, growing degree days, and temperature seasonality refer to the input layers used for the climate zone classification proposed by van Wart et al. (2013); see text for further details. Variability in selected variables across crop × country combinations is provided in Supplementary Figure S5.

Variables	Wheat ETH	Wheat ETH	Maize ETH	Maize ETH	Rice WS PHL	Rice DS PHL	Wheat NLD	Wheat NLD	Wheat NLD	Barley NLD	Barley NLD	Barley NLD
Year	2009	2013	2010	2013	2014	2014	2015	2016	2017	2015	2016	2017
Reported crop yield (t ha^−1^)	1.8	1.8	2.5	2.8	4.1	5.1	8.7	7.2	8.3	6.0	5.6	5.5
Aridity index (×1000, mm mm^−1^)	7.2	7.2	6.9	7.3	12.5	12.5	11.9	12.0	12.0	11.6	11.7	11.8
Growing degree days (×100,°C)	57.6	57.7	68.9	68.8	98.9	99.2	35.0	35.0	34.9	33.6	33.5	33.2
Temperature seasonality (×100,°C)	10.2	10.1	9.4	9.6	10.8	10.7	53.5	53.4	53.6	54.6	54.4	54.3
Seed rate (kg ha^−1^)	190.2	195.9	31.0	31.1	96.5	88.5	198.1	206.1	201.1	152.6	148.1	147.9
N applied (kg N ha^−1^)	47.7	48.9	33.3	41.8	108.7	132.6	201.6	209.1	197.1	96.1	93.7	89.1
P applied (kg P ha^−1^)	19.5	20.4	5.2	4.9	28.7	34.1	33.4	37.7	34.7	12.0	13.1	9.9
Field size (ha)	0.45	0.40	1.46	1.38	1.15	1.22	7.83	7.80	7.72	4.39	4.89	5.12
Number of farms (*n*)	1024	1215	1006	1206	1103	854	131	142	187	71	93	91
Number of fields (*n*)	1201	1440	1613	2095	1103	854	352	399	439	152	199	226

Data for wheat and maize crops in Ethiopia were collected by the Ethiopian Institute of Agricultural Research (EIAR) in collaboration with the International Maize and Wheat Improvement Center (CIMMYT). The “Wheat Adoption and Impact Survey” covered the growing seasons of 2009 and 2013 and was conducted to assess the impact of genetic improvement of wheat in Ethiopia (Jaleta et al., 2019; Fig. 2A). For maize, data were compiled for the growing seasons of 2010 and 2013 from the “Sustainable intensification of Maize-Legume Cropping Systems for food security in Eastern and Southern Africa” (SIMLESA) and “Diffusion and Impact of Improved Varieties in Africa” (DIVA) projects (Jaleta et al., 2018; Fig. 2B). The sampling frame comprised the selection of the main growing districts, followed by a random selection of communities within each district, and by a random selection of households within each community to ensure national representativeness.

Data for rice crops in Central Luzon, Philippines, were collected by the International Rice Research Institute (IRRI) under a project aiming to provide ’Metrics and Indicators for Tracking in GRiSP’ (MISTIG, where GRiSP stands for Global Rice Science Partnership). A three-stage sampling procedure was used to identify the households to be surveyed in the top four rice producing provinces of Central Luzon (Fig. 2C), as explained elsewhere (Silva et al., 2018). The household survey covered the 2013 dry season (DS) and the 2014 wet season (WS) for rice in the region and requested information for the largest rice parcel in each farm. WS rice is the traditional crop in the region, with DS rice made possible through past investments in irrigation.

Data for winter wheat and spring barley crops in the Netherlands were obtained from commercial farm management softwares for crop registration and decision support. No specific sampling frame was used for farmer selection and the spatial distribution of the data thus depended on the geographical distribution of farmers using such softwares. The database covers the main crops and agricultural regions of the Netherlands, but for this study only data for winter wheat and spring barley over the growing seasons of 2015, 2016, and 2017 were used (Fig. 2D). Winter wheat is a main crop cultivated across the Netherlands mostly for animal feed. In contrast, spring barley is a minor crop in the Netherlands, largely cultivated in the Northeast of the country (Fig. 2), and sold for malt to the beer industry.

#### 3.1.2 Predictive and explanatory variables

The final database contained a total of 87 variables. Twenty-two variables were obtained directly from the farm field data: geographic coordinates and 20 other soil and crop management variables were self-reported by farmers. Fifty-four climatic variables and nine soil variables were retrieved from secondary data sources, as described below. The full list and description of the variables are provided in Supplementary Table 1.

Secondary data from open access spatial products were added to the database of farm field data based on the GPS coordinates of the surveyed households. Soil variables were obtained from Hengl et al. (2017) with the purpose to describe soil physical and chemical properties for each farm. Climatic data were obtained from three sources: (1) 19 bioclimatic variables were obtained from Fick and Hijmans (2017), (2) three climate zone variables were obtained from the Global Yield Gap Atlas (GYGA; van Wart et al., 2013), and (3) 54 variables were constructed from daily weather records provided by AgERA5 (Boogaard et al., 2020), considering rainfall data from Funk et al. (2015) for Ethiopia and the Philippines. Bioclimatic variables are biologically meaningful as they represent annual trends, seasonality, and extreme or limiting environmental factors for plant growth. GYGA variables are agronomically meaningful and often used to delineate environments for yield gap analysis. Climatic variables from AgERA5 were computed for the growing season and captured average and extreme weather conditions during the growing seasons surveyed. The length of the growing season was defined based on reported sowing and harvest dates for fields in the Philippines and the Netherlands. Farm-specific sowing and harvest dates were not available for data in Ethiopia so average values per district were obtained through expert knowledge and used to retrieve secondary data.

### 3.2 Partitioning variation in yield

Observed yield variability may reflect different sources of random variation, from non-systematic field-level deviations due to localized heterogeneity in growing conditions or observational error due to systematic differences in locations or seasons. Random effects models, i.e., linear mixed effect models with the intercept as the only fixed term, provide a way to estimate the relative contribution of different spatio-temporal factors to total yield variation. A random effects model was fitted for each crop × country combination considering crop yield as dependent variable. Three nested random spatial effects were included to assess how the spatial structure of the data affected residual variance namely: province, district, and farm for cereal crops in Ethiopia and the Netherlands (districts in the Netherlands were defined based on the postal code of each farm), and province, district, and *barangay* (as only one field per farm was surveyed) for rice in the Philippines. Where possible, the effect of time was accounted for by including an interaction between year and each spatial random effect (i.e., province, district, and farm). This was the case for the models fitted to the data from Ethiopia and from the Netherlands, for which repeated farm observations over time were available. The inclusion of location specific year effects allows the random effects due to location to be separated from the effects of season specific conditions at each location. A large variance component for province, district, or farm/*barangay* indicates there are consistent yield differences within the respective spatial unit. Conversely, a large variance component for province:year, district:year, or farm:year indicates yield differences over time for the respective spatial unit (e.g., the same districts can be high- or low-yielding across different years).

The random effects models were fitted with the *lmer()* function of the *lme4* R package (Bates et al., 2015). For each model, the proportion of variance accounted for by the random effects was defined as the ratio between the sum of the variance of the random variables and the total residual variance, i.e., the sum of residual variance accounted for by the random effects and the residual variance not accounted for by these random variables. The proportion of residual variance explained by each random variable was further assessed relative to the residual variance accounted for by the random effects. A spatial analysis of yield variability was done using variograms fitted with the *variog()* function and using conventional kriging implemented with the *krig.conv()* function of the *geoR* R package (Ribeiro et al., 2020). The spatial analysis yielded no conclusive results, hence data are not shown.

### 3.3 Explaining and predicting yield variability

#### 3.3.1 Random forest models

Random forest is a non-parametric machine learning method known to outperform other algorithms in explanatory and predictive analyses (Nayak et al., 2022a, Breiman, 2001a). Ten random forest models with different types of variables were constructed to explain and predict crop yield (Table 2; see also Supplementary Table 1 for a description of all variables considered in each category). Each model contained either predictive (p), explanatory (e), or both predictive and explanatory variables (pe) from one (climatic, c), two (climatic and soil, cs), or three (climatic, soil, and farm survey, csf) categories. Model 1 (M1*gps*) considered the GPS coordinates of the farms in Ethiopia and in the Philippines or fields in the Netherlands. Models 2, 3, and 4 (M2*pc*, M3*pcs*, and M4*pcsf*) included predictive climatic variables, predictive soil variables, and predictive survey variables added cumulatively to each other, and the GPS coordinates considered in model M1*gps*. Models 5, 6, and 7 (M5*ec*, M6*ecs*, and M7*ecsf*) included, respectively, and added cumulatively to each other, explanatory climatic variables, explanatory soil variables, and explanatory survey variables, plus the GPS coordinates considered in model M1*gps*. Model 8 (M8*pec*) included the GPS coordinates as model M1*gps* plus predictive and explanatory climatic variables. Model 9 (M9*pecs*) builds upon model M8*pec* by adding predictive and explanatory soil variables and, finally, model 10 (M10*pecsf*) builds upon model M9*pecs* by adding predictive and explanatory survey variables, hence comprising all 87 variables.

**Table 2:** Description of the random forest models fitted to explain and predict on-farm yield variability. The full list of variables per category is provided in Supplementary Table 1. Subscript codes: *p* = predictive, *e* = explanatory, *c* = climatic, *s* = soil, *f* = farm survey.

Abbreviation	Model description	Explain	Predict
M1*gps*	GPS coordinates only		
M2*pc*	M1 + predictive climatic variables		
M3*pcs*	M2 + predictive soil variables		
M4*pcsf*	M3 + predictive survey variables		
M5*ec*	M1 + explanatory climatic variables		
M6*ecs*	M5 + explanatory soil variables		
M7*ecsf*	M6 + explanatory survey variables		
M8*pec*	M1 + predictive and explanatory climatic variables		
M9*pecs*	M8 + predictive and explanatory soil variables		
M10*pecsf*	M9 + predictive and explanatory survey variables		

Random forest models were fitted using the *rfsrc()* function of the *randomForestSRC* R package (Ishwaran and Kogalur, 2007) considering *ntree* equal to 1000, and default values for *nodesize* (equal to 5) and *mtry* (equal to one third of the number of variables used for model fitting). Variable importance and goodness-of-fit (using 1:1 scatter plots between observed and predicted crop yield for each farm × year combination) were assessed for model M10*pecsf* fitted to the pooled data. Variable importance refers to the mean decrease in accuracy due to permutation of variables when fitting the model. Statistical metrics were estimated for all ten models as explained in Section 3.3.3.

#### 3.3.2 Cross-validation scheme

Data for each crop × country were partitioned into a training and test data set considering a 70:30 ratio, respectively. Data resampling following this ratio was done for different farms, provinces, or years meaning that, for each crop × country, the training data set comprised 70% of unique field-year combinations or provinces and the test data set comprised the remaining 30% of the field-year combinations or provinces, respectively. Cross-validation over time focused on yield prediction across years not considered during model training rather than on within year explanation or prediction. For cross-validation over time in Ethiopia, data were available for two years only and in that case data for one year were used for model training and data for the other year for model testing and vice-versa. Cross-validation over time in the Netherlands considered all combinations of two years for model training and the remaining year for model testing. The test data set was thus always independent from the training data set in evaluations of model performance. Such data re-sampling scheme allows for testing model performance in predicting crop yield of unknown farms while considering the spatial and temporal structure of the data explicitly. Random forest models were fitted on the training data sets, and these models were then used to predict crop yield in the respective test data sets.

#### 3.3.3 Evaluation of model performance

The coefficient of determination (R^2^) and the Root Mean Square Error (RMSE) were used to evaluate the performance of the fitted models. The R^2^ indicates the proportion of variation in the dependent variable explained by the independent variables. The RMSE measures the difference between the values predicted by the model and the observed values, hence providing a measure of the spread of model residuals. The two metrics were computed for all models fitted to the pooled data and for the train and test data sets in the cross-validation scheme. The R^2^ ranges between 0 and 1, with the latter indicating the model explains all the variation observed in the dependent variable. The model fit was considered excellent if the RMSE was lower than 10%, good if greater than 10% and lower or equal to 20%, fair if greater than 20% and lower or equal to 30%, and poor if greater than 30%.

## 4 Results

### 4.1 Describing on-farm yield variability

**Fig. 3:**
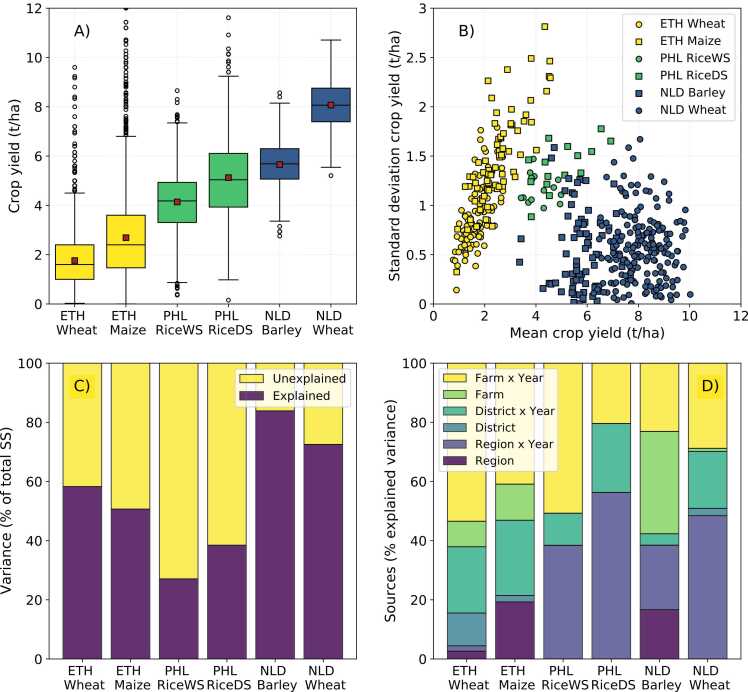
Actual yield variability for each crop × country (A), average and standard deviation of crop yield for a given crop × district × year/season (B), proportion of residual variance accounted for with linear mixed models (C), and variance components for each crop × country (D). In (D), for the Philippines all components are per year, as only one year was available in the data and ‘farm’ effects refer to *barangay* as data were recorded for one field per farm. See text for further explanation. Country codes: ‘ETH’ = Ethiopia, PHL = ‘Philippines’, ‘NLD’ = ‘Netherlands’.

Cereal yields were smallest in Ethiopia, intermediate in the Philippines, and largest in the Netherlands (Fig. 3A). Across the country, wheat and maize yield in Ethiopia were on average 1.8 and 2.7 t ha^−1^ (inter-quartile range equal to 1.4 and 2.1 t ha^−1^), respectively, whereas rice yield in the Philippines was on average 4.1 and 5.1 t ha^−1^ in the WS and DS (inter-quartile range equal to 1.6 and 2.2 t ha^−1^), respectively. Wheat yield in the Netherlands was on average 8.1 t ha^−1^ and that of spring barley 5.7 t ha^−1^, and the inter-quartile range was equal to 1.4 and 1.2 t ha^−1^, respectively. The standard deviation of cereal yield was estimated per administrative unit (the lowest level) in each country × year combination, and ranged between 0.1 and 2.8 t ha^−1^ for cereal crops in Ethiopia, between 0.9 and 1.8 t ha^−1^ for rice crops in the Philippines, and between nil and 1.7 t ha^−1^ for cereal crops in the Netherlands (Fig. 3B). Reported yield was thus least variable, i.e., had a lower standard deviation, for cereals in the Netherlands and for wheat in Ethiopia than for rice in the Philippines, and most variable for maize in certain districts of Ethiopia for which high standard deviations were observed (Fig. 3B).

### 4.2 Partitioning yield variation

The proportion of yield variation accounted for by systematic random effects was 58% and 51% for wheat and maize in Ethiopia, respectively, 27% and 38% for rice yield during the WS and DS in the Philippines, respectively, and more than 70% for cereals in the Netherlands (Fig. 3C). This result indicates that, compared to cereals in the Netherlands, the amount of unexplainable within-farm variation in the lower input systems was substantial, particularly for rice in the Philippines (note this was captured through a random effect of *barangay* for the Philippines as data were available for one field per farm in the respective data set). With respect to the latter, it must be noted that relatively few replicate observations per farm (or *barangay*) were available, which may affect the quality of the estimate for the residual variance.

Farm and farm × year together represented the largest variance components for all crop × country combinations, except for DS rice in the Philippines and wheat in the Netherlands (Fig. 3D), indicating that yield differences at the smallest spatial scale explained most of the systematic variation in crop yield. Conversely, for DS rice in the Philippines and wheat in the Netherlands, the largest variance components were represented by region and/or region × year, indicating greater yield differences at regional level than at the farm level. Moreover, year-specific variance components tended to be larger than time-invariant variance components for the crop × country combinations for which location and year variance components could be separated (Fig. 3D). The only exceptions to this were the large, time-invariant, regional variance components for maize in Ethiopia and the large farm and region variance components for barley in the Netherlands. Indeed stable, not year-specific, region and farm variance components accounted for more than half of the yield variation explained for barley in the Netherlands, which was not observed for any other crop × country combination.

### 4.3 Explaining yield variability

#### 4.3.1 Variable importance

**Fig. 4:**
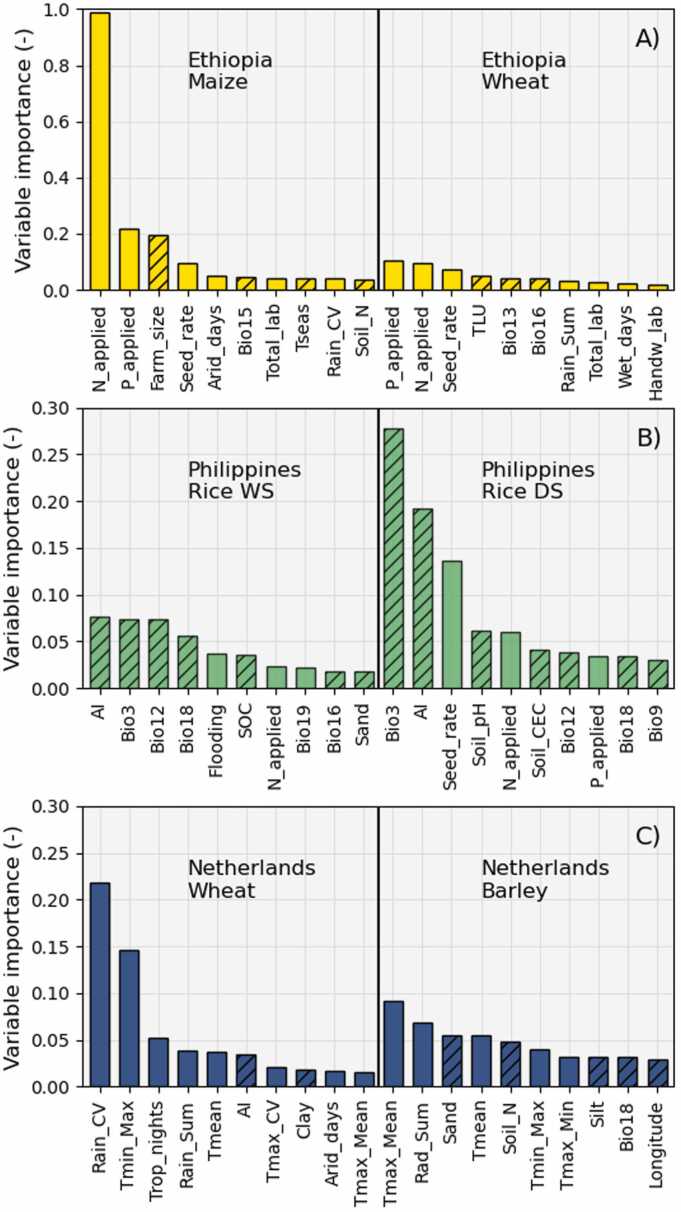
Variable importance of the random forest model M10*pecsf* for wheat and maize in Ethiopia (A), wet season (WS) and dry season (DS) rice in the Philippines (B), and winter wheat and spring barley in the Netherlands (C). Only the top ten most important variables are displayed. Hatched bars show predictive variables whereas non-hatched bars show explanatory variables. See Supplementary Table 1 for an overview of all variables included in the analysis.

In the random forest analysis, management factors were identified as particularly important in explaining yield variability in Ethiopia, whereas yield variability in the Philippines and in the Netherlands was mostly explained by environmental factors (Fig. 4). Predictive climatic variables were important to explain rice yield variability in the Philippines, whereas explanatory climatic variables were most important to explain cereal yield variability in the Netherlands (Fig. 4). The two most important variables explaining maize yield variability in Ethiopia were the amount of N and P applied, followed by the farm size (Fig. 4A). P and N applied were also the most important variables explaining wheat yield variability in Ethiopia, followed by seed rate (Fig. 4A). Aridity index and the bioclimatic variable #3 (isothermality, i.e., the ratio between annual mean temperature and mean diurnal range) were the first and second most important variables explaining WS rice yield variability, whereas the reversed order was true for DS rice (Fig. 4B). The bioclimatic variable #12 (annual precipitation) and seed rate were the third most important variables explaining rice yield variability in the WS and DS, respectively. For winter wheat in the Netherlands, the most important variables explaining yield variability were rainfall variability, the maximum of the minimum temperature registered during the growing season, and the number of tropical nights (number of days with minimum temperature above 20 °C) during the growing season (Fig. 4C). For spring barley, the mean maximum temperature and the cumulative solar radiation during the growing season, and the sand content of the soil were the three most important variables explaining yield variability (Fig. 4C).

The second, third, and fourth most important variables explaining yield variability in Ethiopia and the Philippines became the first, second, and third most important variables when the most important variable shown in Fig. 4 was removed prior to model fitting (Supplementary Figure 2). For winter wheat in the Netherlands, the second and third most important variables became the first and second most important when rainfall variability was removed prior to model fitting, whereas for spring barley the order of the most important variables changed when the mean maximum temperature was removed prior to model fitting (Supplementary Figure 2). These results indicate that the drivers of yield variability are robust and consistent for all crop × country combinations, except for barley in the Netherlands.

#### 4.3.2 Explanatory power

**Fig. 5:**
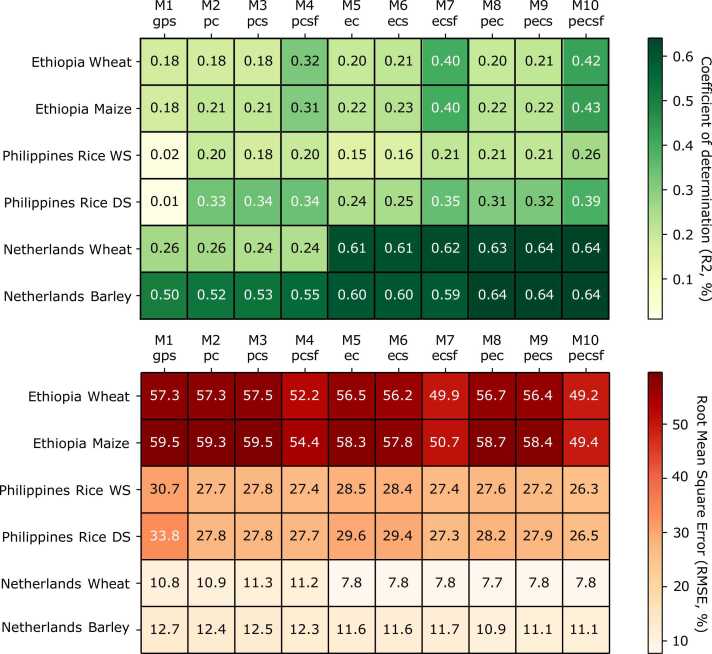
Performance of the fitted random forest models in explaining crop yield variability. The coefficient of determination (R^2^) is displayed in the top heatmap and the RMSE is displayed in the bottom heatmap. See Table 2 for further information about the model codes.

As expected, the random forest model containing all predictive and explanatory variables (model M10*pecsf*), explained the largest proportion of variance and had the lowest RMSE in all cases (Fig. 5). Yet, explanatory power varied quite widely between farming systems. The largest proportion of yield variability was explained for wheat and barley in the Netherlands (64% of variance explained), followed by wheat and maize in Ethiopia (42% and 43%, respectively), and the least for rice in the Philippines (26% and 39% in the WS and DS, respectively; Fig. 5). This result is consistent with the differences in unexplained residual variation observed in the variance component analysis (Fig. 3C). In terms of model accuracy, models of data in Ethiopia performed worse, with an extremely high RMSE, while models for data in the Netherlands showed good accuracy in addition to explaining a high proportion of variance. For all crop × country combinations, model M7 (with explanatory variables only) explained a greater proportion of variance than model M4 (with predictive variables only). The difference in performance between models M7 and M4 was less apparent for data in the Philippines though, where performance was poor for most models. For data in Ethiopia, all models without survey variables performed poorly and were only marginally better than a model with GPS coordinates only, while for data in the Philippines and the Netherlands, adding survey variables hardly improved model performance, with the possible exception for the full model (M10) for DS rice in the Philippines. Soil variables did not improve model performance for any of the farming systems, but for data in the Philippines and the Netherlands adding climatic variables proved essential for explaining additional variation compared to a model with only GPS coordinates. Predictive variables were effective to improve model performance for rice data in the Philippines, in contrast to data for cereals in the Netherlands, for which only adding explanatory (weather) variables improved model performance (Fig. 5).

### 4.4 Predicting yield variability

**Fig. 6:**
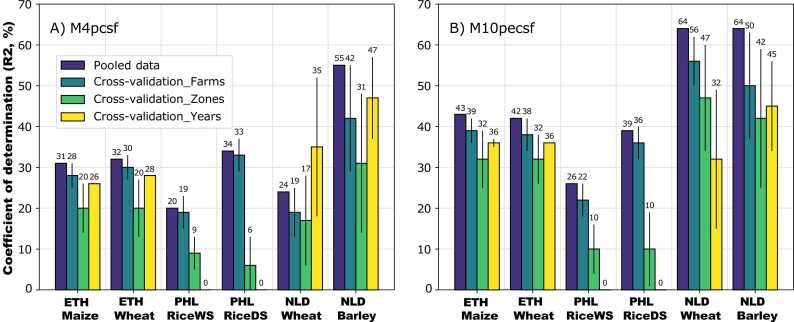
Coefficient of determination (R^2^) of the pooled model (i.e., out-of-bag predictions) and of the models fitted to the test data set in cross-validation runs over farms, provinces, and years. Bars show the mean and error bars the standard deviation across different iterations of the cross-validation scheme with data re-sampling. The full description of the models fitted is provided in Table 2. R^2^ and RMSE values for other models are provided in Supplementary Figures S3 and S4. Country codes: ‘ETH’ = Ethiopia, PHL = ‘Philippines’, ‘NLD’ = ‘Netherlands’.

Model performance, as evaluated above, may provide an overly optimistic idea of the ability of random forest models to explain or predict results at different locations or seasons, which is why cross-validation in space and time is needed. The results of cross-validation in space (Fig. 6) revealed that extrapolation of existing models to newly sampled locations may indeed be problematic, since the proportion of explained variance declined severely when random forest models were cross-validated at a larger spatial scale. This effect was particularly evident for data in the Netherlands where the cross-validation R^2^ diminished steadily from farm to province (zone) to 47% (wheat) and 42% (barley) compared to 64% for the pooled data (Fig. 6, model M10). In relative terms, the reductions in cross-validation R^2^ were even greater for data in Ethiopia and the Philippines, where model performance was poorer to begin with. It should be noted that for data in Ethiopia, fields on the same farm shared the same spatial coordinates and climatic data, which might explain the negligible reduction in model performance when cross-validating the random forest models across different farms.

The results for cross-validation in time were somewhat less clear-cut (Fig. 6). For data in the Netherlands, there was a clear reduction in the cross-validation R^2^ for the models with all variables included (M10), with the reduction in performance being similar to that caused by extrapolating across provinces. For predictive models (M4), the reduction in performance compared to that of the model fitted to the pooled data was relatively minor for barley data in the Netherlands, comparable to the reduction observed in the farm-level cross validation, and non-existent for wheat data also in the Netherlands, although there was considerable uncertainty around the mean values (see error bars in Fig. 6). For data in Ethiopia, model performance was reduced, but remained better than when extrapolating across provinces.

## 5 Discussion

### 5.1 Agronomic interpretation of the results

There were marked differences between the crop × country combinations in terms of average yield and yield variability (Fig. 3A). Both absolute yield variation across all observations as well as variation relative to the mean yield within district × year were notably higher for maize in Ethiopia and rice in the Philippines, particularly when compared to wheat and barley in the Netherlands (Fig. 3B). While part of this may reflect differences in methods of data collection, the greater yield variability of maize than wheat in Ethiopia is consistent with the fact that maize is cultivated across a wide range of agro-ecologies in the country, including lowland areas prone to water stress during the growing season (Abate et al., 2015). In contrast, wheat is mostly cultivated in areas with adequate water supply across the Ethiopian highlands, providing a stable environment for crop production (Schneider and Anderson, 2010). The same is true in the Netherlands, given its humid climate and presence of capillary rise on clay soils (Kroes et al., 2018). An intermediate situation was observed in Central Luzon, Philippines, where lodging of rice panicles is common in the WS (Lampayan et al., 2010) due to heavy rainfall and strong winds from tropical cyclones, whereas the DS provides a more stable environment for rice production provided that irrigation is available (Barker and Levine, 2012).

Partitioning of yield variation showed that spatio-temporal random effects could account for more than 70% of the variance in cereal yield in the Netherlands, ca. 50% of the variance in cereal yield in Ethiopia, and less than 30% of the variance in rice yield in the Philippines (Fig. 3D). This indicates that the contribution of residual, within-farm (i.e., *barangay* for rice data in the Philippines), variation in Ethiopia and the Philippines was substantially larger compared to the Netherlands, perhaps again due to differences in data collection or because of less agronomic homogeneity among fields. Yet, the lowest variance accounted for by random effects for rice in the Philippines might also be explained by the lack of repeated observations over time, which does not allow to assess the contribution of time-varying variance components with the data set used. Spatial-temporal variation was distributed differently among the different data sets. In four out of six cases, most variation was contained at the farm level, as also observed by van Loon et al. (2019) and by van Heerwaarden et al. (2023). Yet, only for the case of barley in the Netherlands, variance in yield was primarily associated with consistent differences among farms across years instead of year-specific differences. The same pattern was observed at higher spatial scales (Fig. 3D). Results for barley might be attributed to the small spatial distribution of the data (Fig. 2D), in other words most farms were located in the same district and region across the different years.

Different groups of variables were identified as important in different farming systems. Firstly, predictive and explanatory farm survey variables improved model performance in Ethiopia (Figure 5), where nutrient application rates were identified as the most important variables explaining wheat and maize yield variability (Fig. 4A). These results corroborate the findings of Silva et al. (2021a) and Assefa et al. (2020) using the same data sets. Secondly, predictive climatic variables alone explained nearly as much yield variability as all the 87 variables taken together for rice crops in the Philippines (Figure 5), with the aridity index and isothermality standing out as important variables (Fig. 4B). Lastly, explanatory climatic variables alone explained nearly as much of the yield variability of wheat and, to a lesser extent, barley in the Netherlands as the full set of 87 variables (Fig. 5). Rainfall variability and minimum temperature during the growing season were important for wheat, whereas mean maximum temperature and cumulative radiation were important for barley (Fig. 4C). These results support earlier studies (e.g., Silva et al., 2020b, Reidsma et al., 2009) pointing to the importance of weather conditions during the growing season in farming systems operating close to yield potential.

Our results are likely affected by the spatial extent of the data set used for each crop × country combination. Clearly, data from Ethiopia covers a much larger geographical area than data from the Philippines and the Netherlands (Fig. 2). Moreover, the smaller spatio-temporal extent of the data set used for rice in the Philippines resulted in slightly smaller variability in some of the spatial covariates used in the analysis, certainly when compared with data for similar variables in the Ethiopia data sets (Supplementary Figure S5). We would expect the spatial extent to matter most for data sets covering large geographical regions with marked differences in environmental conditions. We also expected the latter to be partly captured by GPS coordinates only, as indeed observed for the Ethiopia data (Fig. 5). Yet, for rice crops in the Philippines, predictive climatic variables explain significantly more yield variability than GPS coordinates only (Fig. 5), a result not observed in the other data sets. The latter indicates the importance of fixed climatic conditions (such as aridity index, Fig. 4B), which are not well captured by the GPS coordinates alone. Finally, the relatively high prediction accuracy in the Netherlands is noteworthy, pointing perhaps to better data quality and greater influence of weather conditions than observed in the other data sets.

### 5.2 Explanatory and predictive power

Random forest was proven in earlier studies to be the most suitable method for data-driven agronomy (e.g., Nayak et al., 2022b), which can be attributed to the randomness generated when training the algorithm (Breiman, 2001a). This tree-based algorithm was thus used to test the hypotheses that explanatory variables account for more variation in crop yield compared to predictive variables and that explanatory and predictive power decreases when extrapolating in space and time. Our results indicate that a total of 87 variables dealing with genotype × environment × management interactions (Supplementary Table 1) explained nearly 65% of cereal yield variability in the Netherlands and less than 45% of cereal yield variability in Ethiopia and in the Philippines (Fig. 5), findings which align with the share of residual variance in crop yield explained by random effects models (Fig. 3C). High R^2^ values such as those found for the Netherlands have been reported in other high-yielding cropping systems (Nayak et al., 2022a, Nayak et al., 2022b, Lischeid et al., 2022), but considerably smaller R^2^ have also often been reported in the literature (Tseng et al., 2021, Devkota et al., 2021).

The data sets used (see Section 3.1) differed markedly in the type of variables that contributed most to model performance (Fig. 5). For cereals in Ethiopia, none of the predictive or explanatory climatic and soil variables improved model fit above what was achieved by GPS coordinates alone. Only the addition of survey variables, either predictive or explanatory, increased model performance. Quite the contrary was observed for rice in the Philippines, where GPS coordinates had very little explanatory power, but addition of predictive climatic variables raised R^2^ to above 0.2. The importance of predictive variables for rice in the Philippines did not result in better predictions over space though (Figure 6), probably because the data set covered one single crop year (2013 DS and 2014 WS) and its spatial extent was small (selected areas in Central Luzon only). In the Netherlands, the type of variables included had little impact on model performance, but models containing explanatory climatic variables performed markedly better than those containing only predictive variables. The difference between predictive and explanatory variables was smaller in Ethiopia and the Philippines than in the Netherlands, but in all cases the model containing all predictive and explanatory variables performed best. Improving model performance with additional predictors is thus not straightforward, since observed improvements from additional variables were generally modest (Fig. 5).

Big data from farmers’ fields are useful to explain yield variability to some extent (Fig. 5), but not as much to predict it across space and time, as indicated by a decrease in the cross-validation R^2^ for nearly all crop × country combinations (Fig. 6). Cross-validation against a random subset of farm-year combinations mimics to some extent the bootstrap aggregation method (Breiman, 2001b) used to generate random subsets of data for model training in standard applications of random forest (Tseng et al., 2021, Devkota et al., 2021). Another possible explanation for the small difference in cross-validation R^2^ between these two cross-validation schemes for all crops except barley (Fig. 6) is that fields on the same farm in Ethiopia and the Philippines shared the same spatial coordinates and climatic data. Although random forest is powerful for interpolating data in space and time at regional levels (e.g., Wu et al., 2023, Guilpart et al., 2022), it is less so for on-farm yield prediction across regions and growing seasons not considered for model training (Fig. 6). Our analysis thus demonstrates this limitation of random forest and calls for proper model cross-validation prior to model interpretation and prediction.

Model performance nearly always declined when models were cross-validated in space and time (Fig. 6). Our results thus question the ability of data-driven methods to predict crop yield variability under on-farm conditions even when data sets with a large sample size and number of candidate predictors are available (see also Mulders et al., 2021, Ronner et al., 2016). Cross-validation across provinces reduced model performance independently of the residual variance explained by the random effects (Figs. 3D and 6). Poor cross-validation across provinces is to be expected in data sets with a strong ‘spatial structure’, as captured by large variance components for spatial scales. Our results confirm this for most crop × country combinations, as the largest relative difference in R^2^ between predictions for the pooled data and for cross-validation across provinces was observed for rice in the Philippines, followed by barley in the Netherlands and cereals in Ethiopia, and wheat in the Netherlands (Fig. 6), whereas the relative contribution of region, district, and farm variance components to residual variance decreased in the same order (Fig. 3D).

Strong cross-validation results over time would also be expected in data sets capturing some degree of spatial structure. For cereals in Ethiopia, the R^2^ was fairly low for most models and no substantial decreases in R^2^ were observed when models were cross-validated over time (Fig. 6), probably because a high residual variance was not accounted for by the random effects (Fig. 3C). Cross-validation results across time were somewhat more complex for cereals in the Netherlands. The fairly high R^2^ observed for barley in models with only predictive variables (Fig. 5) confirms the large share of residual variance accounted for by space-dependent variance components (Fig. 3D). Conversely, for wheat, the relatively low R^2^ of models with only predictive variables and relatively high R^2^ of models with both predictive and explanatory variables (Fig. 6) is a result of large time × space interactions in the residual variance (Fig. 6D). Yet, the increase in R^2^ in models with predictive variables only, when cross-validated over time, was unexpected (Fig. 6) and most likely explained by a large variability between random subsets of the data (Fig. 6) and the short time series covered in the data.

### 5.3 Recommendations for data-driven agronomy

The analytical framework adopted here was useful to unpack yield variability and to expose the limits of data-driven crop yield prediction in space and time (Fig. 1). We recommend future studies to (1) adopt cross-validation schemes with data re-sampling explicitly considering the spatio-temporal structure of the data sets at hand, (2) identify the type of variables most valuable to explain and predict crop yield in specific farming systems, and (3) combine data-driven methods with domain knowledge and mechanistic tools (Maestrini et al., 2022). These three steps are essential to better understand on-farm crop yield variability across relatively large scales. They are also important to guide data collection activities in terms of spatial sampling of observational units, required sample sizes, and types of variables needed for sound site-specific agronomic recommendations.

Further investments in data quality are also necessary to improve the performance of data-driven approaches. Errors associated with yield measurements (Kosmowski et al., 2021), farmer recall on field area and input use (Carletto et al., 2013), and inaccuracies in secondary data (Hengl et al., 2017) are known problems of on-farm production data, particularly in low-income countries. We recommend future agronomic diagnostic surveys to measure crop production using crop cuts in different parts of the field and to measure field areas precisely, as already done in some recent applications (e.g., Nayak et al., 2022a,Nayak et al., 2022b, Devkota et al., 2021). Production and area data must be complemented with a minimum set of variables including GPS coordinates, sowing and harvest dates, type of variety, and water management (irrigation vs. rainfed) as these are critical for a detailed characterisation of the biophysical environment where production took place. Other season-specific explanatory variables, and survey variables on management and input use, will also be beneficial to include when explaining yield variability is the aim. Finally, future surveys should be designed according to well-established sampling frames to allow for cross-validation in space, and investments must be made to collect time series data over multiple years for proper model cross-validation in time. This will be critical to unravel the relative contribution of spatial and temporal components to yield variability and hopefully improve the predictive power of data-driven approaches.

## 6 Conclusion

Data is an important asset for agronomic decision making and research in the context of sustainable intensification and digital advisories for farmers. Building upon nearly 11.000 geo-referenced field × year observations across three countries in different stages of agricultural intensification, our results show that cereal yields were less variable in the Netherlands and for wheat in Ethiopia than for rice in the Philippines, and most variable for maize in Ethiopia. A total of 87 variables explained nearly 65% of cereal yield variability in the Netherlands and less than 45% of cereal yield variability in Ethiopia and in the Philippines. Omitting specific groups of variables had a strong impact on model performance, i.e., explanatory crop management variables were most important to explain cereal yield variability in Ethiopia, while predictive climatic variables and explanatory climatic variables were most important to explain cereal yield variability in the Philippines and in the Netherlands, respectively. The R^2^ of the random forest models with only predictive variables declined by 4–28% when these were used to predict cereal yields in provinces or years not considered during model training. A similar decline in model performance (5–32%) was observed for random forest models with both predictive and explanatory variables. Independently of the variables considered and cross-validation scheme used, the explanatory and predictive power of the fitted models was lower for smallholder farms in Ethiopia and the Philippines than for commercial farms in the Netherlands. In conclusion, big data from farmers’ fields is useful to explain on-farm yield variability to some extent, but not to predict it across time and space. Further research is needed to better understand the role of data quality and the spatial and temporal extent of the data sets used to explain and predict on-farm yield variability across large scales, and to critically assess the role big data and machine learning can play on that.

## CRediT authorship contribution statement

**João Vasco Silva:** Conceptualization, Methodology, Software, Formal analysis, Investigation, Data curation, Writing – original draft, Visualization. **Joost van Heerwaarden:** Conceptualization, Methodology, Software, Formal analysis, Writing – review & editing, Supervision. **Pytrik Reidsma:** Methodology, Validation, Resources, Writing – review & editing, Supervision. **Alice G. Laborte:** Validation, Data curation, Writing – review & editing. **Kindie Tesfaye:** Validation, Data curation, Writing – review & editing. **Martin K. van Ittersum:** Methodology, Validation, Resources, Writing – review & editing, Supervision.

## Acknowledgements

We thank Banchayehu Assefa (WUR/CIMMYT) and Moti Jaleta (CIMMYT) for helping with the preparation of the data sets used in the analysis for maize and wheat in Ethiopia, and Ma. Lourdes Velasco (IRRI) for helping with the preparation of the data set for rice in the Philippines. JVS acknowledges the financial support of the WaterFARMING project funded by the Netherlands Science Foundation (NWO; grant agreement ALWWW20161) and of the OneCGIAR Initiative on Excellence in Agronomy supported by the 10.13039/100000865Bill and Melinda Gates Foundation (grant agreement INV-005431). Any opinions, findings, conclusions, or recommendations expressed in this publication are those of the authors and do not reflect the view of the institutions the authors are affiliated to.

## Declaration of Competing Interest

The authors declare that they have no known competing financial interests or personal relationships that could have appeared to influence the work reported in this paper.

## Data Availability

The authors do not have permission to share data.
