# Supplementary material for: Big data, small explanatory and predictive power: Lessons from random forest modeling of on-farm yield variability and implications for data-driven agronomy
Source: Field Crops Res. 2023 Oct 15;302:109063. doi: 10.1016/j.fcr.2023.109063 (PMC10565834; doi:10.1016/j.fcr.2023.109063)
Supplement: Supplementary file 1 — Supplementary material [file mmc1.pdf]

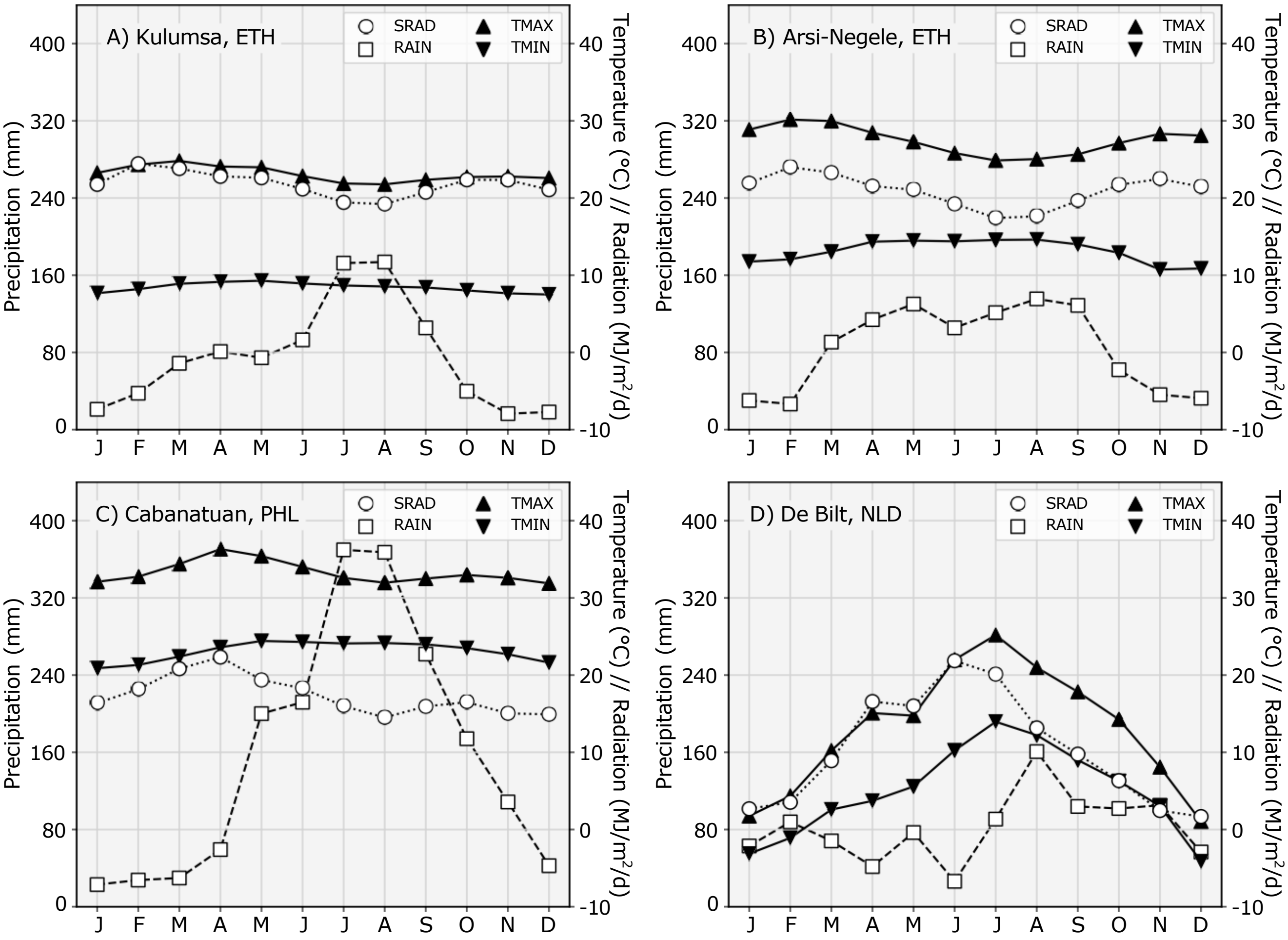

Supplementary Figure 1. Historical weather data for Kulumsa, Ethiopia (A), Arsi-Negele, Ethiopia (B), Cabanatuan, the Philippines (C), and De Bilt, the Netherlands (D).

Supplementary Table 1. Description of the variables used to explain and predict crop yield variability.

**Variable**	**Type**	**Description**	**Unit**
**Climatic variables**			
wc2.1_30s_bio_1	Predictive	BIO1 = annual mean temperature	°C
wc2.1_30s_bio_2	Predictive	BIO2 = mean diurnal range	°C
wc2.1_30s_bio_3	Predictive	BIO3 = isothermality (BIO2/BIO7)	%
wc2.1_30s_bio_4	Predictive	BIO4 = temperature seasonality	°C
wc2.1_30s_bio_5	Predictive	BIO5 = maximum temperature of the warmest month	°C
wc2.1_30s_bio_6	Predictive	BIO6 = minimum temperature of the coldest month	°C
wc2.1_30s_bio_7	Predictive	BIO7 = temperature annual range (BIO5-BIO6)	°C
wc2.1_30s_bio_8	Predictive	BIO8 = mean temperature of the wettest quarter	°C
wc2.1_30s_bio_9	Predictive	BIO9 = mean temperature of the driest quarter	°C
wc2.1_30s_bio_10	Predictive	BIO10 = mean temperature of the warmest quarter	°C
wc2.1_30s_bio_11	Predictive	BIO11 = mean temperature of the coldest quarter	°C
wc2.1_30s_bio_12	Predictive	BIO12 = annual precipitation	mm
wc2.1_30s_bio_13	Predictive	BIO13 = precipitation of the wettest month	mm
wc2.1_30s_bio_14	Predictive	BIO14 = precipitation of the driest month	mm
wc2.1_30s_bio_15	Predictive	BIO15 = precipitation seasonality	%
wc2.1_30s_bio_16	Predictive	BIO16 = precipitation of the wettest quarter	mm
wc2.1_30s_bio_17	Predictive	BIO17 = precipitation of the driest quarter	mm
wc2.1_30s_bio_18	Predictive	BIO18 = precipitation of the warmest quarter	mm
wc2.1_30s_bio_19	Predictive	BIO19 = precipitation of the coldest quarter	mm
ai_1000	Predictive	Aridity index	mm/mm
gdd_100	Predictive	Growing degress day	°C day
temp_seas_100	Predictive	Temperature seasonality	°C
TEMP_cv	Explanatory	Coefficient of variation of mean temperature	%
TEMP_mean	Explanatory	Average mean temperature	°C
TMAX_cv	Explanatory	Coefficient of variation of maximum temperature	%
TMAX_max	Explanatory	Maximum maximum temperature	°C
TMAX_mean	Explanatory	Average maximum temperature	°C
TMAX_min	Explanatory	Minimum maximum temperature	°C
TMIN_cv	Explanatory	Coefficient of variation of minimum temperature	%
TMIN_max	Explanatory	Maximum minimum temperature	°C
TMIN_mean	Explanatory	Average minimum temperature	°C
TMIN_min	Explanatory	Minimum minimum temperature	°C
RAD_sum	Explanatory	Cumulative solar radiation	MJ/m2
RAIN_cv	Explanatory	Coefficient variation of rainfall	%
RAIN_sum	Explanatory	Cumulative rainfall	mm
ET0_cv	Explanatory	Coefficient of variation of reference evapotranspiration	%
ET0_sum	Explanatory	Cumulative reference evapotranspiration	mm
AI_index	Explanatory	Aridity index	mm/mm
arid_days	Explanatory	Number of arid days	#
max_consec_dry_days	Explanatory	Maximum consecutive number of dry days	#

Supplementary Table 1. Description of the variables used to explain and predict crop yield variability (*continued*).

Variable	Type	Description	Unit
wet_days	Explanatory	Number of wet days	#
max_consec_wet_days	Explanatory	Maximum consecutive number of wet days	#
summer_days	Explanatory	Number of summer days	#
max_consec_summer_days	Explanatory	Maximum consecutive number of summer days	#
frost_days	Explanatory	Number of frost days	#
max_consec_frost_days	Explanatory	Maximum consecutive number of frost days	#
heavy_rain_days	Explanatory	Number of days with heavy rains	#
very_heavy_rain_days	Explanatory	Number of days with very heavy rains	#
tropical_nights	Explanatory	Number of tropical nights	#
biol_eff_degr_day	Explanatory	Biological effective degree days	°C day
season_days	Explanatory	Growing season days	#
stress_drought	Explanatory	Drought damage	y/n
stress_flooding	Explanatory	Flooding damage	y/n
stress_typhoon	Explanatory	Typhoon damage	y/n
**Soil variables**			
clay	Predictive	Soil clay content	%
sand	Predictive	Soil sand content	%
silt	Predictive	Soil silt content	%
soc	Predictive	Soil organic carbon	g/kg
ocd	Predictive	Soil organic carbon density	kg/m3
nitrogen	Predictive	Soil total nitrogen	g/kg
cec	Predictive	Cation exchange capacity	$cmol^{+}$/kg
phh2o	Predictive	Soil pH in water	unitless
cfvo	Predictive	Volumetric fraction of coarse fragments	cm3/dm3
soil_conservation	Explanatory	Soil conservation practices	y/n
stress_acidity	Explanatory	Presence of soil acidity	y/n
**Survey variables**			
fsize_ha	Predictive	Field size	ha
sowing_doy	Predictive	Sowing date	DOY
tlu	Predictive	Livestock ownership	TLU
seed_kgha	Explanatory	Seed rate	kg/ha
nfert_kgha	Explanatory	Plant available N applied	kg N/ha
pfert_kgha	Explanatory	Total P applied	kg P/ha
dosage_mm	Explanatory	Irrigation water applied	mm
totlab_persdayha	Explanatory	Total labour use	person-day/ha
handw_persdayha	Explanatory	Total labour use for hand-weeding	person-day/ha
herb_lha	Explanatory	Herbicide application rate	L/ha
herb_yn	Explanatory	Herbicide use	y/n
fung_yn	Explanatory	Fungicide use	y/n
insect_yn	Explanatory	Insecticide use	y/n
mollusc_yn	Explanatory	Molluscicide use	y/n
rodent_yn	Explanatory	Rodenticide use	y/n
pesticide_use	Explanatory	Pesticide use	y/n
stress_disease	Explanatory	Disease incidence	y/n
stress_pest	Explanatory	Pest incidence	y/n
stress_rats	Explanatory	Rodent incidence	y/n
harvest_doy	Explanatory	Harvest date	DOY

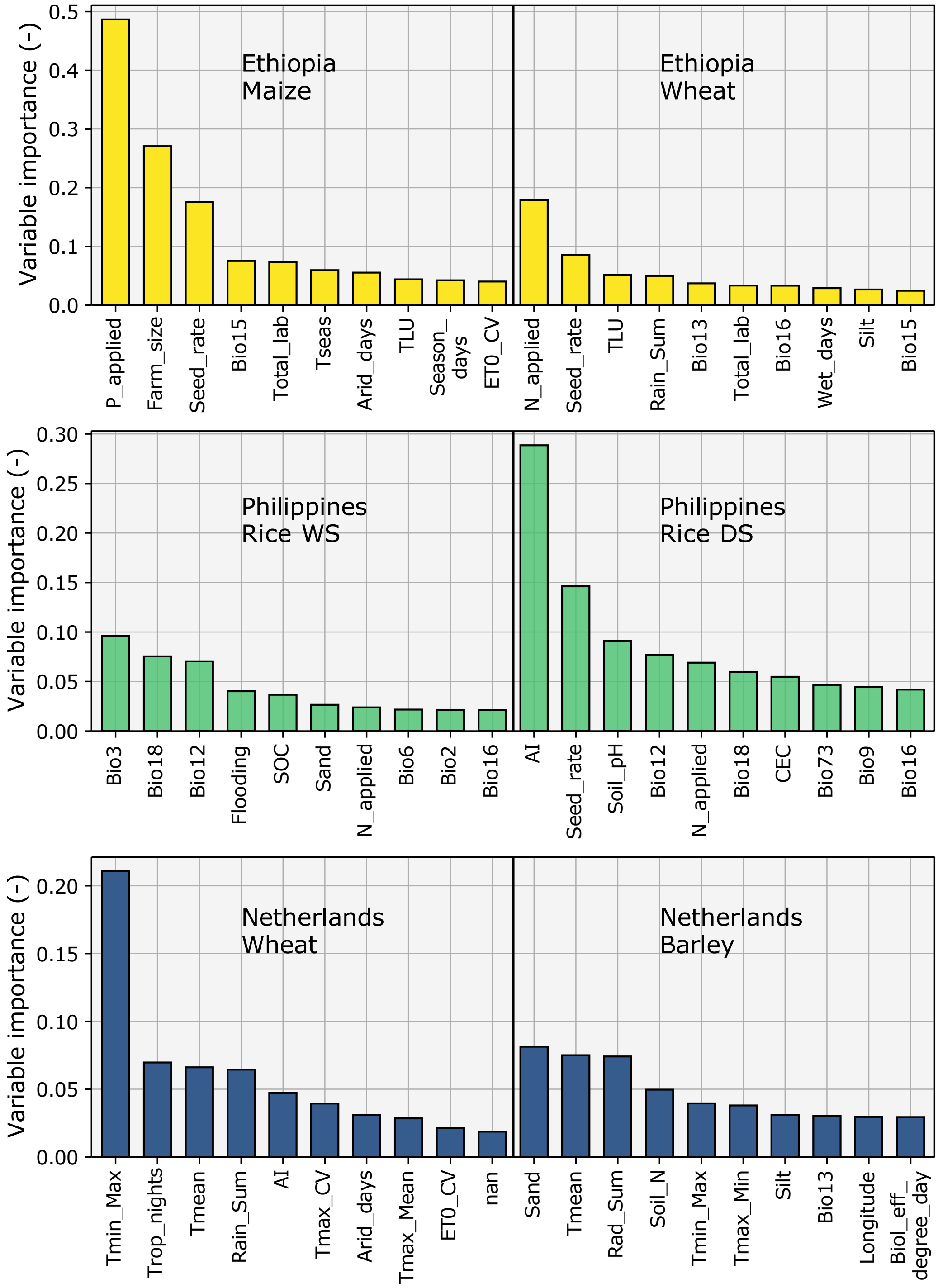

Supplementary Figure 2. Variable importance of the random forest model M10*pecsf* for wheat and maize in Ethiopia (A), wet season (WS) and dry season (DS) rice in the Philippines (B), and winter wheat and spring barley in the Netherlands (C). These models excluded the most important variable reported in Figure 4. Only the top ten most important variables are displayed. See Supplementary Table 1 for an overview of all variables included in the analysis.

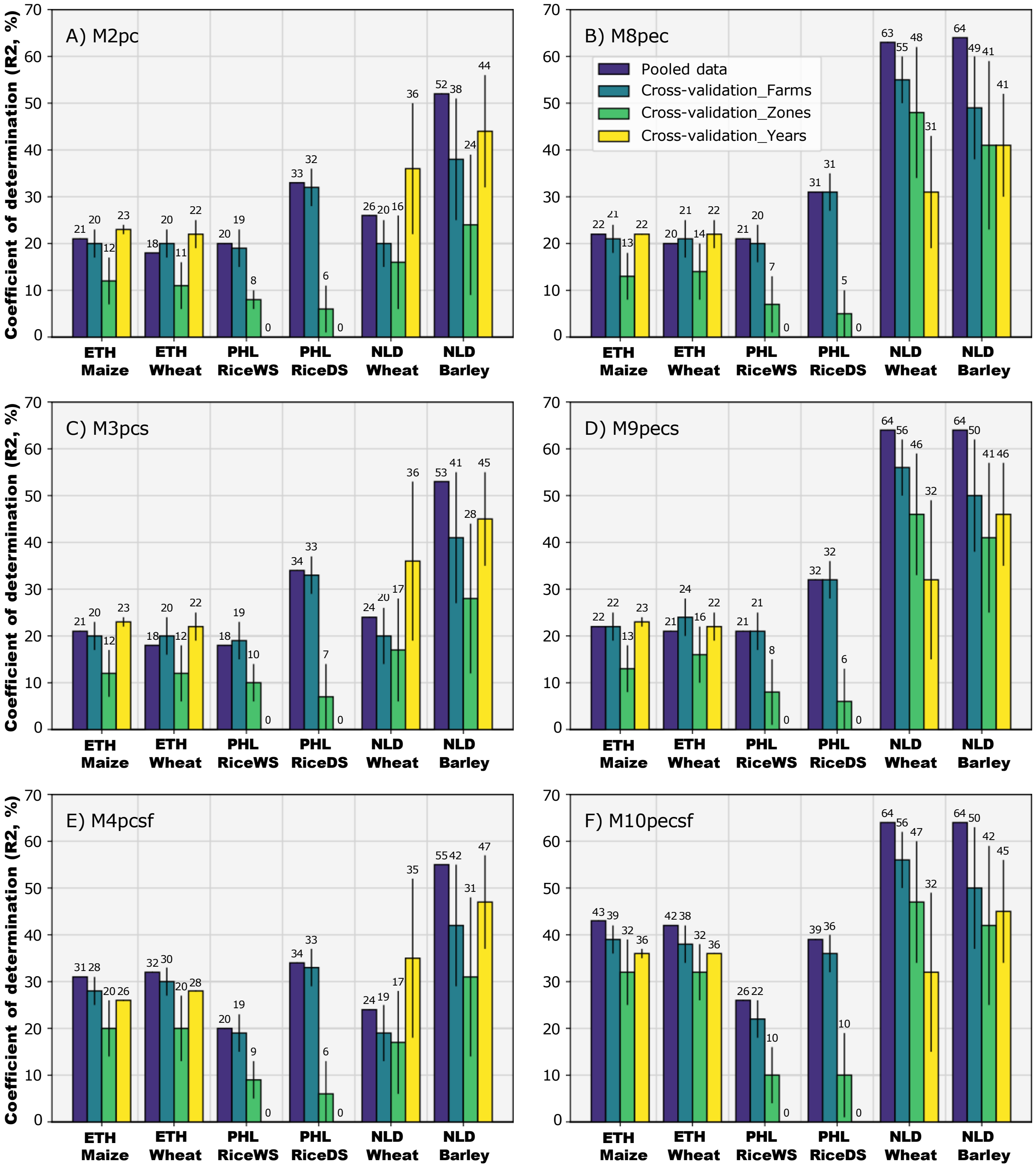

Supplementary Figure 3. Coefficient of determination (R^2) of the pooled model (i.e., out-of-bag predictions) and of the models fitted to the test data set in cross-validation runs over farms, zones, and years. Bars show the mean and error bars the standard deviation across different iterations of the cross-validation with data re-sampling. The full description of the models fitted is provided in Table 2. Country codes: 'ETH' = Ethiopia, PHL = 'Philippines', 'NLD' = 'Netherlands'.

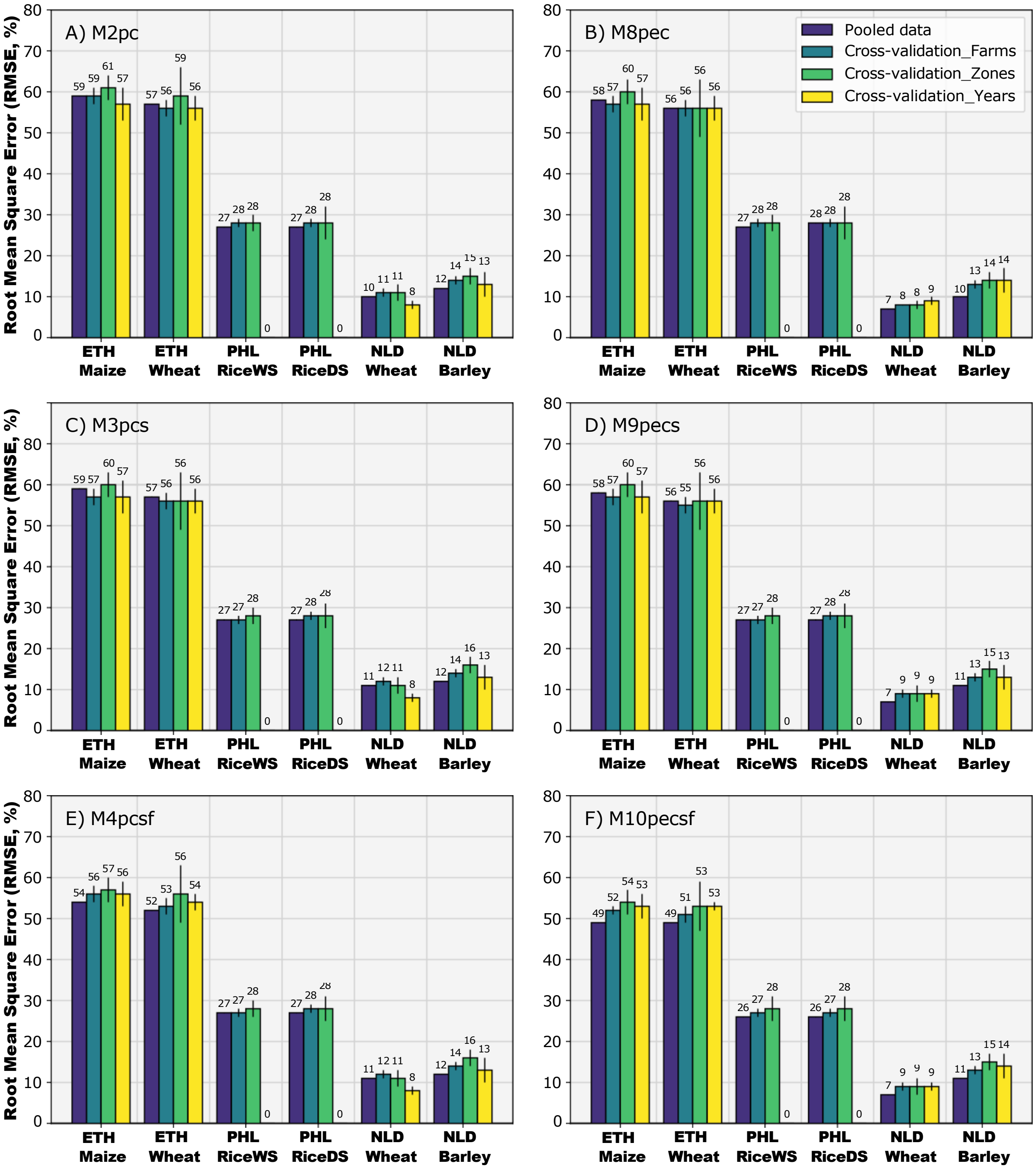

Supplementary Figure 4. Root mean square error (RMSE) of the pooled model and of the models fitted to the testing set in cross-validation runs over farms, zones and years. Bars show the mean and error bars the standard deviation across different iterations of the cross-validation with data re-sampling. The full description of the models fitted is provided in Table 2. Country codes: ’ETH’ = Ethiopia, PHL = ’Philippines’, ’NLD’ = ’Netherlands’.

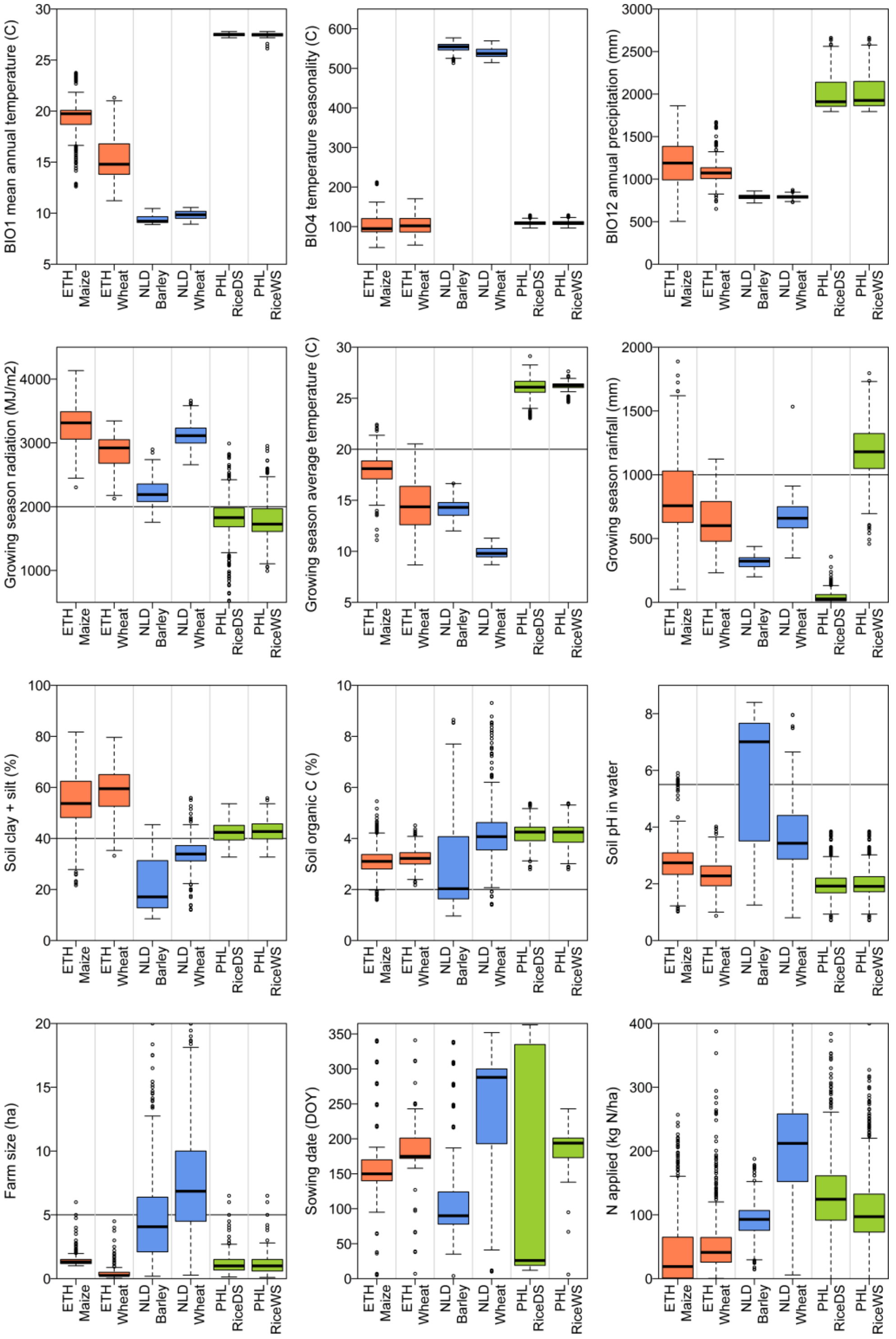

Supplementary Figure 5. Variability in selected predictive climatic variables (first row), explanatory weather variables (second row), predictive soil variables (third row), and predictive and explanatory survey variables (bottom row) across crop × country combinations. Country codes: ’ETH’ = Ethiopia, PHL = ’Philippines’, ’NLD’ = ’Netherlands’.
